# Activity dependent CAM cleavage and neurotransmission

**DOI:** 10.3389/fncel.2015.00305

**Published:** 2015-08-11

**Authors:** Katherine Conant, Megan Allen, Seung T. Lim

**Affiliations:** Department of Neuroscience and Interdisciplinary Program in Neuroscience, Georgetown University Medical CenterWashington, DC, USA

**Keywords:** metalloproteases, MMP, adhesion, CAM, glutamate, dendritic spine

## Abstract

Spatially localized proteolysis represents an elegant means by which neuronal activity dependent changes in synaptic structure, and thus experience dependent learning and memory, can be achieved. *In vitro* and *in vivo* studies suggest that matrix metalloproteinase and adamalysin activity is concentrated at the cell surface, and emerging evidence suggests that increased peri-synaptic expression, release and/or activation of these proteinases occurs with enhanced excitatory neurotransmission. Synaptically expressed cell adhesion molecules (CAMs) could therefore represent important targets for neuronal activity-dependent proteolysis. Several CAM subtypes are expressed at the synapse, and their cleavage can influence the efficacy of synaptic transmission through a variety of non-mutually exclusive mechanisms. In the following review, we discuss mechanisms that regulate neuronal activity-dependent synaptic CAM shedding, including those that may be calcium dependent. We also highlight CAM targets of activity-dependent proteolysis including neuroligin and intercellular adhesion molecule-5 (ICAM-5). We include discussion focused on potential consequences of synaptic CAM shedding, with an emphasis on interactions between soluble CAM cleavage products and specific pre- and post-synaptic receptors.

## A Brief Overview of the Players: Excitatory Synapses, Metalloproteinases, and Cell Adhesion Molecules

### Excitatory Synapses in the Central Nervous System (CNS)

Changes in the number, structure, and/or function excitatory glutamatergic synapses are critical to experience dependent plasticity (Moser et al., 1994; Kopec et al., 2006). In a simplified view, the majority of these synapses are dipartite structures consisting of pre-synaptic axon terminals from which transmitter is released and post-synaptic neurotransmitter receptor-bearing dendritic spines. The latter are small protrusions of varied size and shape that emerge from the dendritic shaft of glutamate-responsive neurons (Alvarez and Sabatini, 2007). Spines with relatively large diameter heads or a mushroom-like morphology tend to be comparatively more stable and to have an increased α-amino-3-hydroxy-5methyl-4-isoxazoleproprionic acid (AMPA) receptor (GluA) number (Matsuzaki et al., 2004; Kopec et al., 2006; Bourne and Harris, 2007; Matsuzaki, 2007; Kasai et al., 2010). Importantly, neuronal activity dependent spine head enlargement, with a concomitant increase in synaptic incorporation of GluAs, is thought to underlie lasting enhancement of synaptic transmission or long-term potentiation (LTP). In a more complex view, glutamatergic synapses can be appreciated as multipartite sites in which glial cell processes approximate pre- and post-synaptic contact sites. Glial cells, or their soluble products, may thus modulate the structural and functional dynamics of neurotransmission (Dityatev and Rusakov, 2011).

### Metalloproteinases in the CNS

Metzincin proteases are zinc-dependent endopeptidases that include cell-secreted matrix metalloproteinases (MMPs) and transmembrane spanning adamalysins [a disintegrin and metalloproteinase (ADAMs)]. These proteases are increasingly appreciated as important effectors of brain function [reviewed in Rivera et al. (2010), Huntley (2012), Sonderegger and Matsumoto-Miyai (2014)]. Though a variety of MMPs and ADAMs are expressed in man, including more than 23 MMPs identified to date (Page-McCaw et al., 2007), it should be noted that a select subset is likely relevant to physiological and pathological CNS plasticity. Family members with well-described expression in neurons, astrocytes, or microglia include MMP-1, MMP-2, MMP-3, MMP-7, MMP-9, MMP-12, MMP-13, MMP-14, and a disintegrin and metalloproteinase-10 (ADAM-10; Yong et al., 2001; Van Hove et al., 2012a).

The cell regulates overall activity of specific MMP and ADAM family members at several levels including that of gene expression. At the transcriptional level, activator protein 1 (AP-1) and nuclear factor kappa-light-chain-enhancer of activated B cells (NF-κB) increase the expression of varied family members such as MMP-9 (Ganguly et al., 2013; see also Table 1 of Berry et al., 2013 for more on transcription factors and select MMPs). In contrast the transcription factor Yin Yang 1 (YY1) directs histone modification to strongly repress transcription of MMP-9 (Rylski et al., 2008). While less is known about the regulation of ADAM family member expression, we do know that the promoter for ADAM10 contains several transcription factor binding-sites including a retinoic acid-responsive element where retinoic acid receptors and retinoic X receptors can bind and thereby activate gene expression (Prinzen et al., 2005; Tippmann et al., 2009). Retinoic acid receptors are present in synaptoneurosomes and dendrites and contribute to select forms of synaptic plasticity (Aoto et al., 2008; Groth and Tsien, 2008).

Post-translational regulation of MMPs and ADAMs is also important with respect to enzymatic activity. Since MMPs typically act on extracellular substrates, release mechanisms represent a potentially important point of control. In a study that activated fibrosarcoma cells with phorbol myristate acetate (PMA), it was shown that subsequent release of MMP-2 and -9 is soluble NSF attachment protein receptor (SNARE) dependent (Kean et al., 2009). The SNARE protein family is critical for calcium-dependent vesicular fusion and release from neurons (Gerber and Sudhof, 2002; Sudhof, 2013). Since neuronal activity can increase intracellular calcium through mechanisms including activation of voltage gated calcium channels, it is tempting to speculate that calcium-dependent MMP release could be facilitated with the same. Of interest, MMP-2 and MMP-9 containing vesicles are observed in the somatodendritic compartment and found in dendritic spines (Sbai et al., 2008; Wilczynski et al., 2008). Moreover, stimuli that may increase intra-neuronal calcium and can induce LTP, can also evoke local MMP-9 release (Wang et al., 2008).

With respect to transmembrane spanning ADAMs, localization is also regulated. For example, ADAM10 and ADAM17 are mainly associated with the endoplasmic reticulum (ER) and Golgi apparatus, with little protein present at the plasma membrane (Schlondorff et al., 2000; Gutwein et al., 2003). ADAM10 contains an ER retention signal at its C-terminus, suggesting that unidentified proteins are required for the ER exit and transport of this protease to the plasma membrane (Marcello et al., 2010, 2012). The binding of tetraspanins to ADAM10 (Xu et al., 2009; Prox et al., 2012) can promote ADAM10 exit from the ER. Synapse-associated protein-97 (SAP-97), a cargo protein involved in protein trafficking at excitatory synapses, can bind to proline-rich sequences in the cytosolic domain of ADAM10 (Marcello et al., 2012). *N*-methyl-D-aspartate receptor (GluN) activation has been shown to affect phosphorylation of SAP97, as well as the transport of ADAM10 from Golgi outposts to synaptic membranes (Saraceno et al., 2014).

The activation of appropriately localized proteases represents an additional point of control. In the case of MMPs, this is typically achieved following release from the cell through cleavage of the pro-domain by other metalloproteinases or plasmin (Nagase et al., 1990). Cleavage of the pro-domain disrupts a critical Cys-Zn^2+^ interaction that otherwise blocks substrate processing (Van Wart and Birkedal-Hansen, 1990). Non-proteolytic activation of MMPs, however, also occurs. For example, nitration or oxidation may alter tertiary structure to activate pro-forms (Gu et al., 2002). Though less well-studied, MMPs may also be active within the cell (Wang et al., 2002).

Finally, MMP activity can be quenched by processes including low density lipoprotein receptor dependent internalization (Hahn-Dantona et al., 2001), and non-covalent interactions with endogenous tissue inhibitors of metalloproteinases (TIMPs; Gardner and Ghorpade, 2003; Visse and Nagase, 2003; Brew and Nagase, 2010).

In terms of brain plasticity related mechanisms that impact MMP expression, release and/or activity, several stimuli or stressors have been studied (see **Table 1** for a partial summary). These include seizure activity (Zhang et al., 1998; Szklarczyk et al., 2002), cytokines (Khuth et al., 2001; Ogier et al., 2005; Ben-Hur et al., 2006), neurotrophins (Kuzniewska et al., 2013), chronic stress (van der Kooij et al., 2014), spatial learning (Wright et al., 2003; Meighan et al., 2006), head trauma (Phillips and Reeves, 2001; Kim et al., 2005), cocaine (Brown et al., 2008; Smith et al., 2014), methamphetamine (Liu et al., 2008), modafinil (He et al., 2011), ischemia (Planas et al., 2001; Rivera et al., 2002), and viral infection of the central nervous system (CNS; Conant et al., 1999; Johnston et al., 2002; Patrick et al., 2002; Zhang et al., 2003). Upregulation of MMP-9 mRNA and enzymatic activity has also been documented in response to neuronal depolarization by KCl (Von Gertten et al., 2003) and kainate (Szklarczyk et al., 2002; Konopacki et al., 2007; Wilczynski et al., 2008; Rylski et al., 2009). MMP-9 levels are increased with LTP (Nagy et al., 2006), and MMP-9 mRNA can be transported to dendrites to undergo local translation and protein release following glutamate stimulation (Dziembowska et al., 2012). Brain-derived neurotrophin factor (BDNF) also upregulates MMP-9 at the mRNA, protein, and enzymatic activity level in dendrites. This process requires engagement of TrkB receptors with subsequent activation of extracellular signal-regulated kinase 1/2 (ERK1/2) and binding of c-Fos to the proximal MMP-9 promoter region (Kuzniewska et al., 2013). In contrast, MMP gene expression can be suppressed by factors including TGF-β, the anesthetic propofol (Zhang et al., 2013), retinoic acid (Osteen et al., 1996; Li et al., 2011; Ye et al., 2011), or sleep deprivation (Taishi et al., 2001).

**Table 1:** Stimuli and stressors linked to altered MMP levels in the CNS.

Effector(s)	MP(s)	Regional focus	Model system	Findings and/or biological relevance	Reference
Seizure induction with kainate	MMP-2 and -9	Hippocampus, striatum, diencephalon, midbrain, frontal cortex, and cerebellum	Rat	(1) Increased MMP-2 and -9 activity (2) Increased GFAP immunoreactivity in hippocampus and frontal cortex	Zhang et al. (1998), Szklarczyk et al. (2002)
Hippocampal dependent learning and memory (Morris water maze)	MMP-3 and -9	Hippocampus and prefrontal cortex	Rat	(1) Increased MMP-3 and -9 protein	Wright et al. (2003), Meighan et al. (2006)
Chronic stress	MMP-9	CA1 region of hippocampus	Rat	(1) Increased MMP-9 (2) Reductions in Nectin-3	van der Kooij et al. (2014)
Traumatic brain injury	MMP-3	Hippocampus	Rat	(1) Astrocyte-derived MMP-3 levels and activity increase 7 days after induction of traumatic brain injury	Kim et al. (2005)
Ischemia	MMP-2 and MMP-9	Hippocampus, lateral cortex, and striatum	Rat	(1) MMP-9 levels increase at 4 h post injury (2) MMP-2 levels peak at 4 days post injury, which corresponds with an increase in reactive microglia and macrophage infiltration	Planas et al. (2001), Rivera et al. (2002)
Viral infection	MMP-2 and -9	Cerebral spinal fluid (CSF)	Human (cerebrospinal fluid)	Damage to the blood–brain barrier (BBB) may facilitate the CNS ingress of monocytes that mediate brain injury. Select MMPs, such as MMP-2 and -9, can reduce BBB integrity	Conant et al. (1999)
Psychostimulants (methamphetamine, cocaine, and modafinil)	MMP-9	Hippocampus, medial prefrontal cortex	Rat	Data suggest that MMP-9 expression may be involved in the addiction phenotype and/or remodeling of the nervous system	Brown et al. (2008), Liu et al. (2008), He et al. (2011)

While a wide variety of studies have examined MMP and ADAM expression in brain or brain-derived cell cultures as a function of specific stimuli, CNS changes have also been examined in a limited number of mutant mouse models. A summary of results from studies using this approach is shown in **Table 2**.

**Table 2:** Altered neuronal migration and/or plasticity in mutant mouse models.

Animal model	Regional focus	Results	Reference
MMP-3 knock out mouse	Cerebellum	(1) Increased size of the external granular layer (EGL) and enhanced granule progenitor cell proliferation at post-natal days 8–12 (2) Delayed migration of granule cells to the EGL (3) Decreased length and complexity of Purkinje cells at post-natal day 12	Van Hove et al. (2012b)
MMP-3 knock-out mouse	Visual cortex (layer V)	(1) Decreased dendritic length and increased number of apical oblique dendrites in pyramidal neurons	Aerts et al. (2014)
MMP-9 overexpressing rat	Hippocampus	(1) Increased length of dendritic spines in CA1	Michaluk et al. (2011)
MMP-9 knock-out mouse	Cerebellum	(1) Increased granule precursor cell number and decreased apoptosis in the external granular layer at post-natal day 12	Vaillant et al. (2003)
MMP-9 knock-out mouse	Hippocampus (slices)	(1) Impaired magnitude and duration of LTP	Nagy et al. (2006)
MMP-2/-9 double knock-out mouse	Nucleus accumbens	(1) Decreased sensitization and reward behavior following methamphetamine (2 mg/kg) administration	Mizoguchi et al. (2007b)
MMP-2 and MMP-9 single knock out mice (observed in both)	Cerebral cortex	(1) Increased number of cell in cerebral-cortical layers 2–3 (2) Altered ICAM-5 and L1CAM levels as a function of age	Tian et al. (2007)
ADAM-10 knock-out mouse	Hippocampus	(1) Decreased neural progenitor cell number with increased differentiation toward the neuronal lineage (2) Impaired performance on a hippocampal-dependent test of memory	Zhuang et al. (2015)
MMP-9/-12 double knockout	Corpus callosum	(1) Decreased myelination at post-natal day 7 (2) Decreased oligodendroglial cell number at post-natal day 10	Larsen et al. (2006)

### CAMs in the CNS

Cell adhesion molecules represent transmembrane adhesion molecules expressed at cell contact sites including the synapse. These molecules typically belong to one of several superfamilies which include cadherins, neurexins/neuroligins, and Ig-domain containing members [reviewed in Benson and Huntley (2012)]. CAMs can mediate stable cell–cell junctions and select family members also play a role in the initiation of synapse formation. Cell surface levels of these molecules, as well as adhesive contact strength, are modified by processes including clathrin-dependent endocytosis (Kamiguchi and Lemmon, 2000). In addition, varied transmembrane CAMs have important intracellular interactions. For example, while integrin cytoplasmic tails do not possess endogenous kinase activity, they interact with critical effectors of intracellular protein phosphorylation cascades (Clark and Brugge, 1995). Finally, through cis-interactions, transmembrane CAMs may influence the localization of synaptic proteins. As a potential example, *N*-cadherin, GluN1 and L1 are found together in large multiprotein complexes (Husi et al., 2000) suggesting that GluN may be part of a membrane adhesion complex (Sheng and Lee, 2000).

Accumulating evidence demonstrates that disrupted CAM expression can influence experience dependent plasticity. For example, ablation of *N*-cadherin from excitatory forebrain synapses of post-natal mice is associated with an alteration in the composition of glutamatergic synapses, so that levels of the GluA1 subunit and PSD95 are diminished (Nikitczuk et al., 2014). Earlier work by the same group has shown that a conditional *N*-cadherin knockout causes a reduction in the maintenance, but not induction, of LTP (Bozdagi et al., 2010). These studies are of particular relevance in that conditional ablation of *N*-cadherin addresses potential confounds that might be associated with knockout effects on early brain development.

Neuron-specific deletion of dystroglycan, a transmembrane protein that links extracellular matrix and the cytoskeleton, also reduces LTP. Specifically, neuron-specific deletion of this protein is associated with a blunting of high frequency stimulation (HFS) induced LTP at CA3–CA1 synapses (Satz et al., 2010). Dystroglycan is expressed by varied cell types including glia, and glial expression of the molecule is involved in forebrain development (Satz et al., 2010).

A number of studies have also investigated LTP in mice that lack specific Ig-domain CAM family members [comprehensively reviewed in Dityatev et al. (2008)]. Neural cell adhesion molecule (NCAM) is a homophilic binding protein that is expressed on the surface of neurons and glia and has been implicated in neurite outgrowth and synaptic plasticity. Indeed, NCAM-deficient mice show impaired LTP in area CA3 (Cremer et al., 1998), and impaired LTP in NCAM knockouts can be rescued by increasing GluN dependent glutamate transmission (Kochlamazashvili et al., 2012). In related work, Cremer et al. (1998) studied mice with a targeted deletion of a polysialyltransferase that attaches polysialic acid (PSA) to NCAM, and that is expressed predominantly in post-natal life (Eckhardt et al., 2000). These animals were shown to have lower post-natal levels of PSA in the brain as well as impaired LTP in CA1 that is evident by 4 weeks of age. Mice that are deficient in ICAM-5, an additional Ig-domain family member expressed on excitatory neurons of the telencephalon (Oka et al., 1990; Benson et al., 1998), also show changes in glutamatergic synapses. These animals show an increase in the dendritic spine/filopodia ratio at P7, suggesting that full length ICAM-5 may delay spine maturation (Matsuno et al., 2006). Though full length ICAM-5 is gradually excluded from spines during their developmental maturation, it remains in approximately 60 percent of spines in adult hippocampal neurons (Sakurai et al., 1998; Matsuno et al., 2006). An antibody directed against ICAM-5, which would presumably disrupt adhesive interactions important to filopodial maintenance, inhibits LTP in rat hippocampus (Sakurai et al., 1998). In mouse hippocampus, however, LTP is relatively increased in an ICAM-5 null animal (Nakamura et al., 2001). Though confounds include antibody specificity, as well as developmental and compensatory effects in the knockout, results are of interest with respect to ICAM-5 as a potential modulator of glutamatergic function.

Specific neuroligin family members have also been investigated with respect to glutamatergic transmission. These are cell adhesion proteins on the post-synaptic membrane that mediate the formation and maintenance of synapses between neurons. Neuroligins act as ligands for β-Neurexins, which are located on the presynaptic membrane. Of particular interest is a study of an autism-associated point mutation in the neuroligin tail that was evaluated following generation of a knock-in mouse (Etherton et al., 2011). Whole-cell voltage-clamp recordings in hippocampal CA1 pyramidal neurons from the knock-in showed a decrease in mini excitatory post-synaptic current (mEPSC) frequency but not amplitude. Changes in GluA receptor subunit composition or presynaptic release possibility were excluded by additional studies, and it was suggested that the neuroligin-3 cytoplasmic tail modulates recruitment of GluAs to post-synaptic sites of excitatory synapses (Etherton et al., 2011).

In addition to *in vitro* and animal model based studies, human genetic studies are consistent with an important role for CAMs in neuroplasticity. Mutations in contactin-associated protein 2, which may promote neuronal circuit assembly during development (Anderson et al., 2012), predispose to autism. Moreover, polymorphisms in CAMs including cadherin 13 (Johnson et al., 2008; Uhl et al., 2014) are associated with addiction risk.

## Synaptic CAMs: Perfectly Poised Substrates for Neuronal Activity Dependent Cleavage

Though a role for CAMs in processes such as LTP could be in whole or large part secondary to the function of full-length molecules, it should also be considered that synaptically localized CAMs represent especially attractive targets for neuronal activity dependent proteolysis. CAM cleavage could disrupt stable interactions with exogenous CAM ligands and/or cause additional effects, including generation of bioactive or dominant negative receptor fragments. Varied CAMs are expressed at synaptic contacts including *N*-cadherin, L1-CAM, ICAM-5, DSCAM, syndecan 2, syncam 2, and neuroligin (Benson et al., 1998; Peixoto et al., 2012; Sonderegger and Matsumoto-Miyai, 2014), and activity-dependent, membrane-proximal cleavage of these molecules is supported by *in vitro* studies that have demonstrated juxtamembrane shedding for specific family members (Peixoto et al., 2012; Sonderegger and Matsumoto-Miyai, 2014). Data from analysis of cerebrospinal spinal fluid samples also supports shedding of CAMs (Strekalova et al., 2006). In this case, shed CAMs likely access the interstitial space from where they in turn gain access to CSF. In one study, an increase in levels of soluble ICAM-5 ectodomain was detected in the CSF of patients with epilepsy and/or infection (Lindsberg et al., 2002; Tian et al., 2008). In related work, N terminal sequencing of NCAM fragments from the CSF of patients with schizophrenia was performed and a disease-associated increase in levels of ectodomain fragments observed (Vawter et al., 2001).

Regulated cleavage of synaptic CAMs can influence glutamatergic transmission through several non-mutually exclusive mechanisms including reduced synaptic stability, conversion of N-terminal CAM ectodomains into soluble effectors of plasticity, and increased generation of intracellular domains (ICDs) that influence transcription. With respect to ICD generation, it should be noted that ectodomain shedding of CAMs is frequently followed by intramembranous gamma secretase cleavage to generate specific C terminal fragments [reviewed in Jordan and Kreutz (2009)].

### Activity Dependent Cleavage of Neuroligin and *N*-Cadherin

Though it has been suggested that ectodomain shedding is highly regulated with only 2% of cell surface proteins released by this process (Hayashida et al., 2010), emerging evidence suggests that neuronal activity dependent CAM shedding represents an important mechanism by which synaptic structure and function are modulated. For example, neuronal activity dependent cleavage of neuroligin-1 is triggered by GluN activation and dependent on MMP or ADAM activity (Peixoto et al., 2012; Suzuki et al., 2012). It occurs in a membrane proximal location and results in destabilization of neuroligin-1’s presynaptic partner, neurexin-1β. Destabilization of neurexin is in turn thought to reduce the probability of presynatic neurotransmitter release (Peixoto et al., 2012).

GluN agonists, as well as ADAM and MMP family members that are regulated in a neuronal activity dependent manner, have also been linked to *N*-cadherin shedding (Reiss et al., 2005; Uemura et al., 2007; Williams et al., 2010; Paudel et al., 2013; Porlan et al., 2014). One of many potential sequelae of this event is the associated generation of a C terminal fragment which is quickly processed by gamma secretase to generate a smaller intracellular fragment that destabilizes a protein critical for CREB dependent transcription (Marambaud et al., 2003).

### Activity Dependent Cleavage of Ig-domain CAMs

In work related to a potential role of ICAM-5 shedding in developmental plasticity, it has been shown that long term treatment (16 h) of DIV 14 hippocampal neurons with 5 μM NMDA or AMPA stimulated an MMP-dependent increase in supernatant levels of shed ICAM-5. ICAM-5 is expressed on dendritic elements of excitatory/spiny neurons in the telenchephalon (Benson et al., 1998). Since full length ICAM-5 may be a negative regulator of filopodia-to-spine transition (Matsuno et al., 2006), these findings are consistent with the possibility that ICAM-5 shedding contributes to developmental spine maturation.

Studies related to the possibility that ICAM-5 cleavage may occur in a relatively rapid manner to influence activity dependent glutamatergic transmission in the adult CNS have also been performed. NMDA stimulation of cultured hippocampal neurons and high frequency tetanic stimulation of hippocampal slices have both been linked to relatively rapid MMP-dependent ICAM-5 shedding (Conant et al., 2010b). In cultured cells, appreciable release of soluble ICAM-5 into culture supernatants can be detected within 5 min of NMDA exposure (Conant et al., 2010b).

Additional studies have examined neuronal activity dependent cleavage of nectin-1, an Ig-like adhesion molecule expressed at puncta adherentia junctions in the CA3 pyramidal region of adult mouse hippocampus (Lim et al., 2012). Of interest, *in vitro* over-expression of cleavage resistant mutants of nectin 1 is associated with an increase in the density of dendritic spines (Lim et al., 2012). One possibility is that cleavage resistant mutants might lead to an increase in the stability of spines.

Elegant work on a related adhesion molecule, demonstrated enhanced MMP-9 dependent cleavage of nectin-3 in perisynaptic CA1 in the setting of chronic stress (van der Kooij et al., 2014). Intriguingly, inhibition of MMP-9 activity or GluN activation led to a reduction chronic stress related behavioral alterations.

IgLON family members, abundant GPI anchored transmembrane proteins, are also processed in a metalloproteinase dependent manner. The IgLON family is a subgroup of the immunoglobulin superfamily cell adhesion molecules (CAMs) and composed of limbic system-associated protein (LAMP), opioid binding cell adhesion molecule (OBCAM), neurotrimin (NTM) and Kilon. Long term treatment of hippocampal neurons with a broad spectrum MMP inhibitor and subsequent pull down of surface proteins demonstrated that inhibitor-treated neurons show increased levels of specific IgLON family members including NTM (Sanz et al., 2014).

Glutamate and MMP dependent shedding of synaptic cell adhesion molecule 2 (SynCAM-2) has also been described (Bajor et al., 2012), which is of interest given the role of this molecule in synapse organization and function (Biederer et al., 2002; Fogel et al., 2007).

### Emerging and Future Studies of Neuronal Activity Dependent CAM Cleavage

Matrix metalloproteinases and ADAMs can also act on a variety of less traditional CAMs including nerve-glia antigen 2 (NG2), β-dystroglycan, and amyloid precursor protein (APP) and netrin-G ligand-3 (Ahmad et al., 2006; Michaluk et al., 2007; Lee et al., 2014; Sakry et al., 2014). There is evidence that these molecules are shed in a neuronal activity-dependent manner, and that they play a role in developmental and/or adult plasticity. For example, recent work suggests that glutamatergic transmission is altered in NG2 knockout animals (Sakry et al., 2014). Future studies will be necessary to further explore mechanisms by which shedding of these proteins can influence plasticity.

Future studies will also be necessary address issues related to shedding of dimers versus monomers, as well as issues of whether single nucleotide polymorphisms (SNPs) influence shedding. Results from recent work suggest that ADAM dependent shedding of neuregulin-1 requires prior dimerization (Hartmann et al., 2015), and analysis of soluble ICAM-1 in pleural fluid suggests that this molecule may also be shed as a dimer (Melis et al., 2003).

Future studies could additionally explore the question of whether post-translational modifications such as glycosylation can influence the cleavage and/or bioactivity of protease-generated CAM fragments. And finally, unbiased proteomics could be utilized to examine interactions between shed CAMs and other proteins in the background of select physiological and pathological processes.

## Metalloproteinases and Synaptic Transmission

Despite their ability to stimulate effects that could both enhance or depress neurotransmission, the majority of studies support a view in which non-pathological neuronal activity stimulates an MMP dependent enhancement of long term memory and its correlates. For example, several groups have demonstrated that MMP inhibitors reduce LTP stimulated by HFS and/or theta burst stimulation (TBS; Nagy et al., 2006; Meighan et al., 2007; Conant et al., 2010b). Inhibition of MMP activity also reduces chemical LTP (cLTP) associated increases in the firing rate and bursting of dissociated cultures of primary hippocampal neurons (Niedringhaus et al., 2012).

Consistent with their effects on hippocampal LTP, varied biochemical and behavioral studies support a role for MMPs in hippocampal dependent learning and/or memory. For example, knockout of MMP-9 impairs contextual fear conditioning (Nagy et al., 2007). Interestingly, in wild-type animals contextual fear conditioning increases hippocampal MMP-9 protein levels as well as MMP-9 dependent cleavage of dystroglycan (Ganguly et al., 2013). These data suggest that MMP-9 plays a role in hippocampal memory association and/or retention. It has also been shown that hippocampal MMP-3 and -9 mRNA levels are increased with Morris water maze (MWM) training, as are levels of active MMP-3 and -9 protein (Meighan et al., 2006). Moreover, treatment with the non-competitive GluN antagonist, MK801, reduces training-associated increases in specific MMP levels, as well as post-training performance assessed by latency to reach platform. Intra-hippocampal or intra-cerebral ventricular injection of a broad-spectrum chemical MMP inhibitor, as compared to artificial CSF control injection, can also reduce time spent in the target quadrant during the MWM probe trial (Meighan et al., 2006). Of interest with respect to anesthetic-modulation of learning and memory, it has been shown that while MWM training can induce a gradual increase in pro- and active-MMP-9, propofol can reduce this increase and also disrupt spatial memory retention 24 h after training (Zhang et al., 2013). In contrast, the wake promoting agent modafinil increases MMP-9 expression in dorsal hippocampal CA3 in a model of REM sleep deprivation (He et al., 2011). In this same model, modafinil increases synapsin 1 expression in an MMP-9 dependent manner. In addiction-related plasticity work, it has been shown that context dependent learning of nicotine induced conditioned place preference (CPP) is associated with an increase in hippocampal MMP-2, -3, and -9 expression, and that exposure to a chemical MMP inhibitor during nicotine induced CPP training can block CPP acquisition (Natarajan et al., 2013). In addition, methamphetamine-induced behavioral sensitization is reduced in mice lacking MMP-2 or MMP-9 (Mizoguchi et al., 2007b).

Matrix metalloproteinase activity can also contribute to enhanced glutamatergic transmission in regions including striatum and amygdala. For example, a chemical MMP inhibitor can disrupt reconsolidation of a fear memory associated with a conditioned stimulus that is independent of contextual cues (Brown et al., 2009). In studies with MMP-9 null mice, Kaczmarek and colleagues have shown that MMP activity in the central amygdala is required for appetitive but not aversive learning (Knapska et al., 2013). In recent work related to cocaine and MMP levels in nucleus accumbens core, an increase in gelatinase activity as detected by *in situ* zymography was detected along neuronal soma and dendrites (Smith et al., 2014). AMPA/NMDA ratios were also increased in medium spiny neurons in cocaine extinguished rats and further increased by cue-induced reinstatement, in an MMP dependent manner. MSNs also showed MMP dependent changes in MSN spine head diameter and/or number in cocaine extinguished and reinstated animals (Smith et al., 2014).

## Mechanisms by which MMPs Influence Neurotransmission; A Focus on CAM Cleavage as a Means to Generate Integrin-Binding Ligands

In terms of the mechanisms by which MMPs modulate actin and spine dynamics to enhance glutamatergic transmission, it should be noted that despite their potential to act on varied substrates such as proneurotrophins (Lee et al., 2001), evidence suggests that their ability to enhance LTP is β_1_ integrin dependent (Nagy et al., 2006; Meighan et al., 2007). Since changes in the number and size of dendritic spines are thought to underlie LTP, Huntley and colleagues (Wang et al., 2008) also monitored spine size and EPSPs simultaneously in hippocampal neurons with combined 2-photon time-lapse imaging and whole-cell recordings. These investigators observed that persistent spine enlargement and synaptic potentiation required both MMP activity and β_1_ integrins (Wang et al., 2008). In related studies, we have previously shown that a β_1_ integrin blocking antibody prevents cLTP associated increases in the overall firing rate of hippocampal-derived neurons (Niedringhaus et al., 2012).

Integrin signaling, and signaling through β_1_ containing integrins in particular, has been well-associated with changes in dendritic and spine actin dynamics (Huang et al., 2006; Shi and Ethell, 2006). The majority of hippocampal integrin heterodimers contain a β_1_ subunit (Pinkstaff et al., 1998; Chan et al., 2006; Huntley, 2012). In addition, β_1_ integrins are expressed on dendrites (Mortillo et al., 2012) and implicated in the regulation of post-natal dendritic arbor and synapse density (Warren et al., 2012). Antagonism of β_1_ integrin signaling by function blocking antibodies or GRGDS peptide administration is associated with a decay of LTP, and hippocampal infusion of function blocking antibodies to β_1_ blocks formation of long-term object location memory (Babayan et al., 2012). Integrin like kinase is also involved in the induction and maintenance of cocaine sensitization, and its silencing prevents sensitization-associated serine-845 phosphorylation of GluA1 (Chen et al., 2010). Though a role for integrin signaling has been implicated in potentiated synaptic activity (Bernard-Trifilo et al., 2005; Nagy et al., 2006; Meighan et al., 2007), the identity of physiologically relevant ligands and important mechanisms by which these ligands are generated has yet to be fully explored.

Recent work is consistent with the possibility that MMP-dependent CAM shedding represents a potential mechanism by which excitatory transmission generates integrin-binding ligands. As opposed to larger ECM components, CAM ectodomains may be relatively soluble. Indeed, previous studies have shown that cleavage of relatively large molecules does not necessarily disrupt three dimensional integrity of the same (Huganir and Racker, 1980). Numerous studies, including those that have demonstrated the integrity of CAM N-terminal fragments in spinal fluid and tissue, support the concept of ectodomain solubility and stability (Lindsberg et al., 2002).

**FIGURE 1:**
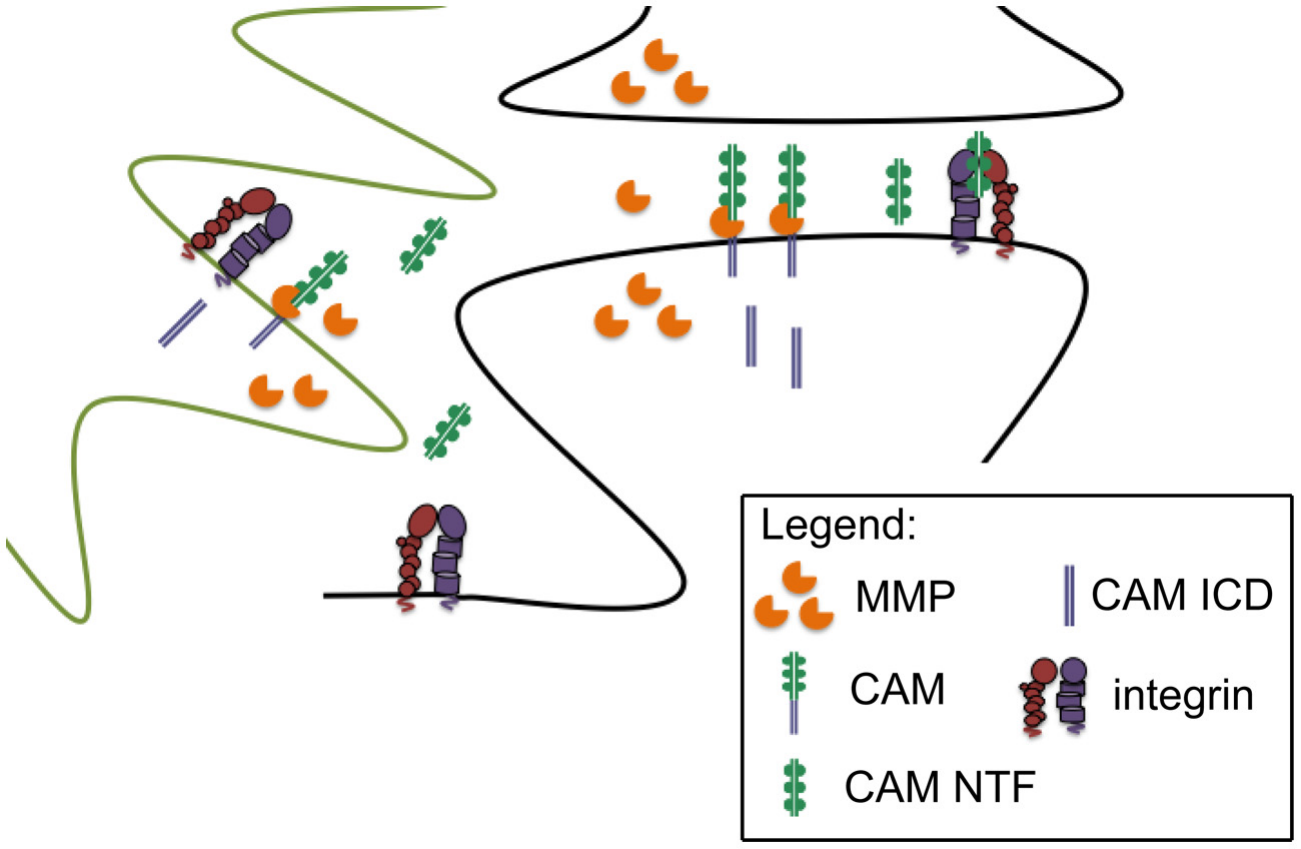
**Synaptically localized CAM cleavage.** Pre and post synaptic components (black outline) and glial components (green outline) of the synapse are shown. MMPs can be released from neurons and/or glia to cleave peri-synaptic CAMs, thus generating CAM N-terminal fragments with the potential to stimulate intregrin dependent signaling. Though not the focus of this review, cleavage generated CAM intracellular domains (ICDs) may also influence synaptic transmission.

Cell adhesion molecule ectodomains are known to possess integrin-binding motifs and indeed stable CAM/integrin interactions have been described (Conant et al., 2010a; Kelly et al., 2013; Ning et al., 2013). CAM ectodomains can also stimulate integrin dependent signaling. For example, shedding of the L1 adhesion molecule has been shown to stimulate integrin dependent cell migration (Mechtersheimer et al., 2001). In addition, we have observed that soluble ICAM-5 can stimulate a β_1_ dependent increase in action potential frequency in cultured hippocampal neurons (Niedringhaus et al., 2012). We also observe co-immunoprecipiation of both full length and shed N terminal ICAM-5 with β_1_ in hippocampal lystates from methamphetamine challenged mice, suggesting that the shed ectodomain may interact with β_1_ *in vivo* (Conant et al., 2010a). NCAM and NG2 can also interact with integrins, and though not yet tested for effects on integrin-dependent neurotransmission, dorsal hippocampal injection of PSA-NCAM has been shown to partially restore impaired contextual memory in NCAM deficient mice (Senkov et al., 2006). Of interest, mice that overexpress the NCAM ectodomain show memory impairments that are similar to those observed in the knockout (Pillai-Nair et al., 2005). This is consistent with a dominant negative effect as well as the possibility that dysregulated ectodomain shedding is deleterious, and it underlines our need to better explore the bioactivity of CAM ectodomains in both physiological and pathological conditions. A schematic of CAM ectodomain shedding from glia and/or neurons, with subsequent integrin binding, is shown in **Figure 1**.

**FIGURE 2:**
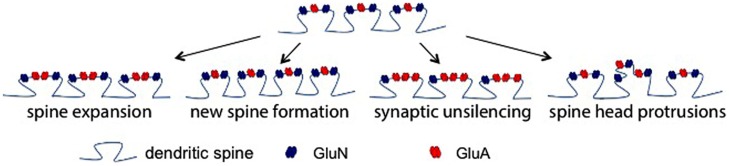
**Post-synaptic mechanisms by which MMP activity could enhance glutamatergic transmission.** There are several non-mutually exclusive possibilities by which MMP activity could influence excitatory neurotransmission. Shown (top) is a dendritic segment with representative spines. As the schematic suggests with arrows to show potential changes, existing spine expansion, new spine formation, synaptic unsilencing, and formation of spine head protrusions represent post synaptic changes that might contribute to MMP and/or integrin dependent changes in the post synaptic element.

In terms of the functional consequences of MMPs and/or CAM ectodomains at single synapses to in turn affect LTP, several non-mutually exclusive possibilities exist as shown in **Figure 2**. In the first, supported by high resolution imaging (Wang et al., 2008), MMP activity causes a change in actin dynamics with a subsequent widening of spines. This would likely bring an increase in functional GluA receptors to the spine head and increase amplitude of mEPSCs. A second possibility is that MMP generated integrin binding ligands could stimulate the growth of new spines. Integrin signaling has been linked to the same (Shi and Ethell, 2006), and though we did not observe a significant increase in spine number in ICAM-5 ectodomain stimulated DIV 14 rat hippocampal neurons at 1 or 24 h (Lonskaya et al., 2013), it would be premature to rule out the possibility that this measure could be increased at other time points or following exposure to additional CAM ectodomains. The potential for integrin binding ligands to cause an unsilencing of post-synaptic components that were previously silent due to deficient synaptic levels of GluA receptors should be considered as a third possibility. Integrin signaling can activate protein kinases that would in turn phosphorylate specific GluA subunits to enhance their synaptic entry (Lim et al., 2008; Chen et al., 2010). Consistent with this possibility, in previous work we have observed both an ICAM-5 ectodomain stimulated increase in the phosphorylation and membrane localization of GluA1, and an increase in the frequency of mEPSCs (Lonskaya et al., 2013). A fourth possibility is that MMP-dependent signaling stimulates in increase in spine head protrusions to affect glutamatergic transmission. While LTP is generally thought to represent an experience dependent increase in dendritic number and/or size (Kopec et al., 2006), increased complexity of spines might also occur. It has been shown that MMP-9 can stimulate an increase in spine head protrusions (Szepesi et al., 2013). This finding is of significance in that these protrusions may be PSD-95 and GluA positive (Richards et al., 2005; Szepesi et al., 2013), and they may be functionally active in terms of mediating glutamateric neurotransmission (Richards et al., 2005).

Future studies related to CAM-integrin interactions will be necessary to examine a variety of additional questions including that of which integrin binding ligands are generated with learning and memory *in vivo.* Further study of whether ectodomain shedding plays and important role in select MMP dependent endpoints including changes in spine size or number (Shi and Ethell, 2006; Wang et al., 2008), the development of spine head protrusions (Szepesi et al., 2013), and developmental changes in neurite outgrowth and dendritic arbor (Van Hove et al., 2012a) may also be warranted. MMPs have also been linked to changes in neuronal excitability (Wojtowicz and Mozrzymas, 2014), and since integrin signaling can also influence ion channel function (Wildering et al., 2002), this could represent a parallel topic for future exploration.

An additional avenue for exploration includes the question of whether the downstream effects of CAM shedding can synergize with events that follow from MMP-dependent processing of additional synaptic substrates. As an example, we consider protease activated receptor-1 (PAR-1). A select subset of MMPs target PAR-1, a G protein coupled receptor that is activated by cleavage in N-terminal domain and consequent exposition of a tethered peptide ligand (Vergnolle et al., 2001; Soh et al., 2010). The receptor is expressed on select neuronal subpopulations and has been detected in synaptoneurosomes (Han et al., 2011; Maggio et al., 2013a,b). While activation of neuronal PARs has the potential to enhance GluN subunit phosphorylation and GluN function (Gingrich et al., 2000), whether PAR activation enhances neuronal integrin signaling is unknown. In non-neural cells, however, it has been shown that PAR-1 activation can enhance integrin affinity for ligands (Shattil et al., 2010).

## CAM Shedding: From Physiology to Pathology

While regulated physiological release of MMPs can contribute to adaptive plasticity, it is important to note that dysregulated release has the potential to disrupt the same (Wojtowicz and Mozrzymas, 2014). Consistent with this, MMP inhibitors have been shown to ameliorate neuronal injury in a number of disease models. In many of these studies, however, reduced MMP-dependent damage to blood brain barrier integrity is likely to account for much of the observed neuroprotection (Asahi et al., 2001). There is, however, an increasing appreciation synaptic proteolysis in particular as significant a contributor to neurological disease. To follow, we will briefly discuss two disorders in which altered synaptic proteolysis may be critical to disease expression. The first is fragile X syndrome (FXS) and the second is psychostimulant addiction.

### Fragile X Syndrome

Fragile X syndrome is a leading genetic cause of intellectual disability and autism (Santoro et al., 2012). At present, no cure is available. Symptoms include developmental delay and increased susceptibility to seizures, while pathological findings include relatively dense and immature dendritic spines (Galvez and Greenough, 2005; McKinney et al., 2005; Pan et al., 2010; Santoro et al., 2012). Expansion of the trinucleotide CGG repeat in excess of 200 repeats located in the 5′ untranslated region of the X chromosome-linked *FMR1* gene cause FXS (Verkerk et al., 1991; Sutcliffe et al., 1992). This leads to transcriptional silencing and a consequent lack of functional protein product, fragile X mental retardation protein (FMRP). FMRP regulates expression of a subset of dendritically localized mRNAs, and thus levels of dendritically localized proteins may be altered in FXS (Darnell et al., 2012; Santoro et al., 2012). FMRP generally acts to inhibit the translation of target genes, but in select cases it may have actions that lead instead to enhanced translation (Darnell et al., 2012; Santoro et al., 2012). For example, FMRP is thought to enhance translation of superoxide dismutase (Bechara et al., 2009), and thus antioxidant enzyme activity might be reduced in FXS. This has implications for glial activation, which has been observed in association with the disease (Rossignol and Frye, 2012). Moreover, neuronal and/or glial expression of MMPs may be elevated in response to increased glial activation and/or oxidant stress (Gu et al., 2002).

Published studies have linked MMP activity to FXS. Levels of MMP-9 are increased in affected humans and in a murine model (Sidhu et al., 2014). Increased dendritic translation of MMP-9 has also been observed (Janusz et al., 2013). Recent work suggests that expression of additional MMPs may be increased as well. For example, transcripts for MMP-2, -3, -7, -9, and -24 are increased in heavy polysomes from *Fmr1* mutant mice (Gkogkas et al., 2014). In terms of functional consequences, dendritic spine abnormalities in a mouse model of FXS can be reduced by minocycline, an inhibitor of MMP activity that can access the brain (Bilousova et al., 2009). Spine abnormalities are also reduced in the background of MMP-9 deficiency (Sidhu et al., 2014). Moreover, exogenous MMP administration to cultured neurons has been associated with relevant changes in spine morphology (Bilousova et al., 2006). Interestingly, MMP knockout can also reduce neuronal circuit defects in a drosophila model of disease (Siller and Broadie, 2011).

The mechanism(s) by which excess MMP activity stimulates the FXS phenotype have yet to be determined. Several possibilities have been suggested, including increased signaling by MMP-dependent activation of pro-neurotrophins and/or generation of integrin-binding laminin fragments (Sidhu et al., 2014). It is tempting to speculate, however, that excess generation of integrin-binding CAM fragments might play a role. Future studies to address this question may therefore be warranted.

### Addiction

Matrix metalloproteinase-dependent changes in synaptic structure and function are also thought to contribute to the maladaptive learning and memory associated with addiction to stimulants including methamphetamine. Methamphetamine is a widely abused illicit drug that has high addictive potential. A variety of studies suggest that methamphetamine is linked to metabolic changes in the brain as well as to synaptic injury (Pu et al., 1996; Volkow et al., 2001; Chang et al., 2009). Evidence for increased MMP expression, release, and/or activation in the setting of methamphetamine exposure comes from several groups. This stimulant can increase release of MMP-1 from cultured neural cells (Conant et al., 2004). This observation is consistent with results from rodent studies in which methamphetamine stimulates increased binding of AP-1, a transcription factor critical to the expression of MMPs including MMP-1 (Akiyama et al., 1996). Additional studies have demonstrated that 5 days of exposure to the drug (2 mg/kg/day) is associated with increased MMP-2 and -9 protein in the frontal cortex and nucleus accumbens of rats (Mizoguchi et al., 2007a). Moreover, an acute high dose of MA (40 mg/kg) is followed by increased mRNA expression of MMP-9 in murine CNS (Liu et al., 2008). Similarly, cocaine, which is similar to methamphetamine in its potential to increase catecholamine levels, has been shown to increase MMP-9 activity in the medial prefrontal cortex at 1, 3, and 24 h post-administration (Brown et al., 2008).

Methamphetamine has the potential to increase MMP expression through several non-mutually exclusive mechanisms including increased catecholamine dependent signaling, activation of glutamate receptor signaling, and increased oxidant stress. For example, methamphetamine associated increases dopamine can act on D1 type dopamine receptors to enhance substrate proteolysis (Iwakura et al., 2011). Since both D1 and D2 type are linked to βγ subunits that can activate PKC and release of intracellular calcium, activation of either receptor type might stimulate calcium dependent MMP release and/or PKC dependent activation of a transmembrane MMP. Another possibility is that MA increases levels of glutamate, as has been shown by Yamamoto and colleagues (Mark et al., 2004), and that glutamate signaling can in turn stimulate increased MMP expression and/or activity. Relatively high concentrations of MA also stimulate an increase in signaling by reactive oxygen intermediates (Lee et al., 2002), which can enhance both the expression and the activation of select MMPs (Gu et al., 2002).

Increased MMP activity may also contribute to synaptic and behavioral changes observed with stimulant exposure. It has been shown that methamphetamine-induced behavioral sensitization is reduced in mice lacking MMP-2 or MMP-9 (Mizoguchi et al., 2007b). Protease activity has also been shown to contribute to cocaine associated CPP (Brown et al., 2007). In recent work focused on structural and function changes at the levels of the synapse in the setting of cocaine exposure paradigms, an increase in the AMPA/NMDA current ratio was increased in extinguished rats, and further increased 15 min following cue-induced reinstatement (Smith et al., 2014). An increase in the AMPA/NMDA ratio after extinction was restored to control by a selective MMP-2 inhibitor, while the altered reinstatement ratio was restored by either and MMP-2 or -9 inhibitor (Smith et al., 2014). Complementary measures of spine density and spine size supported a view in which MMP-2 could increase the density and head size of spines in extinguished animals and that MMP-9 activity contributed to an increase in spine size with reinstatement.

Overall, studies related to the role of MMPs in addiction are exciting and should stimulate further work to address underlying mechanistic components.

## Summary

Matrix metalloproteinases were named for their ability to cleave extracellular matrix proteins such as laminin and collagen. While matrix remodeling effects may be essential during development and wound healing, proteolysis of cell surface receptors including CAMs could represent a critical means by which MMPs can fine tune synaptic structure and function in a more stable or relatively mature CNS. This possibility is supported by work showing that neuronal activity stimulates proteolysis of synaptically localized CAMs, and that CAM shedding can influence varied measures of synaptic transmission. Future studies will be necessary to examine CAM cleavage as affected by variables including cell type, brain region, stimulus type/duration, and developmental stage. Future studies will also be necessary to determine which CAM cleavage products are most likely to influence MMP dependent plasticity *in vivo*, and to determine the extent to which CAM shedding combines with additional MMP-stimulated events to influence experience dependent plasticity.

## Conflict of Interest Statement

The authors declare that the research was conducted in the absence of any commercial or financial relationships that could be construed as a potential conflict of interest.
